# Editorial: Fatigue: physiology and pathology

**DOI:** 10.3389/fnins.2024.1368897

**Published:** 2024-02-02

**Authors:** Sławomir Kujawski, Paweł Zalewski, Lynette Hodges, Jo Nijs, Julia L. Newton

**Affiliations:** ^1^Department of Exercise Physiology and Functional Anatomy, Ludwik Rydygier Collegium Medicum in Bydgoszcz Nicolaus Copernicus University in Toruń, Bydgoszcz, Poland; ^2^Laboratory of Centre for Preclinical Research, Department of Experimental and Clinical Physiology, Warsaw Medical University, Warsaw, Poland; ^3^School of Sport, Exercise and Nutrition, Massey University, Palmerston North, New Zealand; ^4^Pain in Motion International Research Group, Department of Physiotherapy, Human Physiology and Anatomy, Faculty of Physical Education and Physiotherapy, Vrije Universiteit Brussel, Ixelles, Belgium; ^5^Chronic Pain Rehabilitation, Department of Physical Medicine and Physiotherapy, University Hospital Brussels, Brussels, Belgium; ^6^Department of Neuroscience and Physiology, University of Gothenburg, Gothenburg, Sweden; ^7^Retired, Newcastle upon Tyne, United Kingdom

**Keywords:** ME/CFS, chronic fatigue, Multiple Sclerosis, fatigue, long COVID, PACS

## Introduction

Most of us perceive a rich amount of mind states throughout our lives. A qualia could be a derivate of an amalgamation of multiple qualities at a given moment. The severity of fatigue is like a wool coat that is worn by the mind. When the severity of fatigue is low, most healthy people are unaware of its presence. However, when fatigue severity increases, the wool coat gets wet and its presence becomes more and more unpleasant and overwhelming. In pathological conditions, the heaviness of the coat of the fatigue can significantly limit a patient's functionality.

A clear distinction between mind and body appears to be abandoned by the neuroscience paradigm of today at the expense of the underlying interconnection between those two dimensions (Glannon, 2020). Nevertheless, even with the clear advancement of nervous system imaging tools in recent decades, there are potent barriers to translating neural system activity to mental states and experiences, including fatigue (Gonzalez-Castillo et al., 2021). We hope that the current Research Topic provided a closer inspection of one of the fabrics making up the mind.

## Fatigue physiology: prevalence, assessment, stigma

In the review on global fatigue prevalence in the general population included in the Research Topic, ninety-one studies involving 623,624 participants were taken into account (Yoon et al.). Authors observed that 6% of minors and 15% of adults worldwide report fatigue, with a tendency to higher rates in women vs. in men (Yoon et al.). Ten percent of adults and one-and-a-half percent of minors experience chronic fatigue. In addition to having a frequency that is greater in women than in men, medically unexplained exhaustion is also 2.7 times more common than explained fatigue (Yoon et al.).

von dem Knesebeck and Barbek reported data gathered from one thousand two hundred and nine responders regarding public stigma toward individuals with fatigue. Results showed that a significant amount of responders are prone to apply stereotypical labels to people with fatigue, such as being “hypersensitive” or characterized by “weak will” (von dem Knesebeck and Barbek).

Modern brain imaging techniques offer an opportunity to gather huge amounts of data. It seems that neural correlates of fatigue are highly complex (Sun et al., 2014). Therefore, one can facilitate the process of finding patterns in data by applying mathematical models. Yuan et al. applied a variety of methods including the attention mechanism and the gated recurrent unit for fatigue detection using electroencephalography (EEG).

## Fatigue pathology: MS, ME/CFS, long COVID

It should be noted that in some circumstances, acute fatigue seems to be a physiological response to a higher workload. In healthy people, fatigue can be alleviated by rest in a rather short matter of time. However, in patients with Myalgic Encephalomyelitis/Chronic Fatigue Syndrome (ME/CFS), an increase in symptom severity can be caused by relatively low intensity physical, emotional, or psychological stressors. Post-exertional malaise (PEM) is a crucial source of suffering in the majority of ME/CFS patients. PEM is comprehensively described in the paper by Vøllestad and Mengshoel.

Core symptoms of ME/CFS also often occur in long COVID (otherwise known as post-acute sequelae of COVID-19) patients, and may include fatigue, pain, post-exertional malaise, breathing difficulties, and cognitive dysfunction (Davis et al., 2021; Komaroff and Bateman, 2021). In the current main paradigm of science, it is assumed that symptoms perceived by patients are output products of the nervous system. However, there is still much to explore and the study of Thapaliya et al. seems to be a very important step in this direction. “Pain” and “breathing difficulty” perceived by patients were related to multiple brain regions, including pons, midbrain, and whole brainstem volumes (Thapaliya et al.).

The last decades of research in human neuroscience have produced an appreciation of bidirectional cooperation between the nervous system and its effectors. In this line, Day et al. have provided a study on the relationship between changes in the cardiovascular system response to changing position from prone to supine with cognitive dysfunction in patients suffering from ME/CFS and long COVID.

Fatigue is a non-specific symptom, occurring in multiple chronic disorders. In addition, to the description of fatigue in Multiple Sclerosis (MS), Pinarello et al. described modes of treatment of MS both pharmacological and non-pharmacological including telemedicine approach.

## Fatigue management: breathing exercises and integrative care

In skeletal muscle physiology, peripheral fatigue refers to a group of changes occurring in response to prolonged muscle activity, while central fatigue is the inability of the nervous system to produce and transmit the signal to contract to the effectors (Dotan et al., 2021). This distinction is somewhat controversial, as there seems to be no clear barrier between “peripheral” vs. “central” (Dotan et al., 2021). Nevertheless, in keeping with this distinction, Amiri and Zemková suggested study protocol for the application of a breathing exercise program in decreasing fatigue of postural skeletal muscles.

By taking a whole picture of all manuscripts published on the above Research Topic, one can appreciate the multidimensionality of research on fatigue. Because of the innate complexity of physiology and pathology in various conditions, and interaction with the ambient world, it is worth looking at the current medical system in Western countries from various perspectives. A piece of this approach has been implemented in the paper by Araja et al..

In conclusion, the Research Topic includes several important scientific contributions to the field of fatigue physiology and pathology. This includes work addressing the prevalence, assessment, stigma, and fatigue pathology in patients with MS, ME/CFS, and long COVID, as well as reports on fatigue management. Although the field of fatigue physiology and pathology is a growing field, many research gaps remain. Therefore, the guest editors call for more cutting-edge research to advance this field of research, especially to improve care for millions of people suffering from severely debilitating ME/CFS.

## Author contributions

SK: Writing—original draft, Writing—review & editing. PZ: Writing—original draft, Writing—review & editing. LH: Writing—original draft, Writing—review & editing. JN: Writing—original draft, Writing—review & editing. JLN: Writing— original draft, Writing—review & editing.
